# Stroke in Saudi Arabia: a review of the recent literature

**DOI:** 10.11604/pamj.2014.17.14.3015

**Published:** 2014-01-15

**Authors:** Asirvatham Alwin Robert, Marwan Mohamed Zamzami

**Affiliations:** 1 Research Center, Medical Affairs, Sultan Bin Abdulaziz Humanitarian City, Riyadh, Saudi Arabia; 2 Department of Orthopedic Surgery, College of Medicine, King Saud University, Riyadh, Saudi Arabia

**Keywords:** Stroke, Saudi Arabia, disability

## Abstract

Stroke is a major cerebrovascular disease resulting in high mortality and persistent disability in adults across the world. Besides coronary heart disease and cancer, stroke is the commonest cause of death in most industrialized countries. Survivors of stroke are often left with severe mental and physical disabilities, which create a major social and economic burden, ranking as the second most common cause of death worldwide and a major source of morbidity. The Kingdom of Saudi Arabia (KSA) is the largest country in the Middle East occupying approximately four-fifths of the Arabian Peninsula supporting a population of more than 28 million. Stroke is becoming a rapidly increasing problem and an important cause of illness and deaths in Saudi Arabia. However, compared with the developed countries, research regarding the incidence, prevalence and their socio-demographic properties of stroke is still insufficient due to lack of appropriate studies being conducted in these specified areas. This review aims to discuss the range of the aspect of stroke in Saudi Arabia from the literature published.

## Introduction

A stroke or Cerebro-Vascular Accident (CVA) involves the rapid loss of brain function caused by a disruption of blood supply to the brain. Triggered by ischemia (lack of blood flow) or blockage (thrombosis, arterial embolism) or a hemorrhage [1], stroke has become one of the leading causes of serious, long-term neurologic impairment and functional disability and is the cause of mortality globally. However, there are no known drug therapies to improve recovery after stroke. Depending on the severity and type, stroke can leave an individual with a residual damage of physical, psychological, social and cognitive functions [1–4]. The established risk factors, including arterial hypertension, diabetes mellitus, cigarette smoking, micro-vascular rupture, hyperlipidemia, age and observed comorbidity, such as sickle cell disease, human immunodeficiency virus/acquired immune deficiency syndrome infection and cerebral malaria are increasingly being encountered in the tropics [5, 6]. According to the World Health Organization (WHO), around 15 million people, the world over, suffer from stroke each year. Among these, 5 million die and another 5 million are permanently disabled. Four out of five strokes occur in the low and middle income countries who can least afford to manage with the consequences of this disease. However, if nothing continues to be done, the predicted number of people who will die from stroke will increase to 6.7 million each year by 2015 [7]. It is therefore, important to note that while the incidence of stroke is dropping in the West, it is probably ominously increasing in Asia [8]. Research has shown that the Middle East region faces a double burden of the disease due to decreasing rates of communicable diseases and the growing rates of non-communicable diseases [9]. Stroke is increasingly emerging as a major health problem, with the projection that mortality resulting from it will nearly double by 2030, in this region [10]. On the other hand, a study reported that a major percentage of the patients had not even heard the term “stroke” in the Gulf Cooperation Council (GCC) countries. Knowledge regarding stroke was poorest among the groups that belonged to the highest risk bracket for stroke [11]. Arab countries constitute populations with a similar lifestyle and diet that may influence stroke risk, type, and survival after stroke, as well as other features similar to the Western and Oriental populations [5]. The Kingdom of Saudi Arabia (KSA) is the biggest country in the Arabian Peninsula, extending over an area of 2,150,000 km^2^ and boasting a population of more than 28 million [12]. Stroke is being observed as a rapidly growing problem and an important cause of illness and death in Saudi Arabia. Therefore, it becomes one of the most imperative social and economic medical issues in the Kingdom [13]. However, compared with the developing countries, the dearth of research currently available on the incidence and prevalence as well as the socio-demographic properties of stroke certainly warrants concern, particularly the lack of appropriate studies in this specified area. In this review, we discuss the range of various aspects of stroke in Saudi Arabia from the literature published.

## Methods

A literature search was conducted with the assistance of a senior researcher. The archives of the National Library of Medicine (PubMed) including the Ovid Medline databases were searched. General search engines were also used to access non-peer reviewed professional and specialist guidelines and workshops on stroke websites. The search was limited to the English and Arabic languages. The articles were selected by reviewing their titles and abstracts with additional references identified from the reference lists of selected articles.

## Current status of knowledge

### Incidence, prevalence and type of stroke

The global burden of stroke is already high and rising, including the growing incidence, mortality, Disability-Adjusted Life Years (DALYs) and economic impact, specifically in the low- and middle-income countries [14, 15]. In Saudi Arabia, the incidence and prevalence of strokes were low when compared with those recorded in the Western countries, which could be because of the predominance of the younger age groups in this region [16–18]. Thus far, no nationwide, if any, research has been conducted recently on the incidence and prevalence of strokes in Saudi Arabia. However, over the past decade there was one study which reported that the crude incidence rate for first-ever incidence of stroke in Saudi Arabia was 29.8/100,000/year [19]. They also reported that ischemic strokes (69%) predominated and Sub-Arachnoid Hemorrhage (SAH) was extremely rare (1.4%)^19^. Between 1982 and 1992, in a study conducted by Al Rajeh et al., in a hospital that exclusively treated the Saudi Arabian National Guard community rated the crude annual incidence rate at 43.8 per 100,000 [17]. Sayed et al., stated that the most frequent among the stroke subtypes were the ischemic infarcts (79%), of which 46% were lacunar infarcts, followed by intracerebral hemorrhage (18.8%), and SAH (2.2%) [20]. Awada et al., reported that ischemic strokes accounted for 76% of the cases, of which one-third were lacunar infarcts. Most of the hemorrhagic strokes were Intra-Cerebral Hemorrhages (ICHs) and only 2% of all strokes were SAHs [21]. Similar results from other studies confirmed that the frequency of ischemic stroke was higher when compared with the other types [22, 23].

A study conducted by Yaqub et al., (1991) on 200 Saudi stroke patients found that cerebral infarction constituted 87% of the strokes, subarachnoid hemorrhage 4.5%, cerebral hemorrhage 6.5% and venous infarction 2%. The vessel most frequently involved was a portion of or the entire middle cerebral artery, which constituted 52% of the arterial infarcts [24]. Lacunar infarcts were observed in 21% of the patients with arterial infarcts, hypertension was noted in 41% of the patients with arterial infarcts and 62% with cerebral hemorrhages [24]. The highest incidence of hypertension was the risk factor among those with lacunar infarcts at 81%, ganglionic cerebral hemorrhages at 80% and infarcts of the deep branches of the middle cerebral artery (57%). Embolic infarcts due to rheumatic heart disease constituted 11% of all arterial infarcts [24].

### Risk factors

Studies reported that old age, high blood pressure, prior stroke or Transient Ischemic Attack (TIA), diabetes, high cholesterol, tobacco smoking and atrial fibrillation were the major risk factors for stroke [25–27]. High blood pressure is the most important modifiable risk factor of stroke [26]. A study reported that the risk factors significant for stroke in the Saudi population are systemic hypertension (38%), diabetes mellitus (37%), heart disease such as atrial fibrillation, ischemic heart disease, valvular disease, cardiomyopathy (27%), smoking (19%) and family history of stroke (14%) [19]. From among the various treatable risk factors, hypertension was found to be the most important risk factor for stroke among the Saudi population [17]. Another study described some of the common risk factors of stroke, which were hypertension associated with diabetes mellitus (40.4%), hypertension alone (24.9%), diabetes alone (11.6%), atrial fibrillation (5.8%), other cardiac factors (5.5%), Transient Ischemic Attack (TIA) and prior stroke (2.1% each), and smoking (1.8%) [20]. Awada et al., reported that hypertension (52%) was the most important risk factor to induce stroke, followed by diabetes mellitus and cardiac disorders in the Saudi population. Further, the frequent causes of cerebral infarcts found were atherosclerosis 36% followed by hypertensive and/or diabetic arteriolopathy 24% and cardiac embolisms 19%. Hypertensive arteriolopathy accounted for two-thirds of the cerebral hemorrhages, whereas strokes related to small artery disease, i.e. lacunar infarcts and ICHs, accounted for 47% of the cases [21]. It becomes evident that the major predisposing factors identified were hypertension followed by diabetes mellitus, cardiac disease and cigarette smoking. Apparently, the combination of hypertension and diabetes mellitus carried a higher, risk especially in women [17, 22, 28]. Moreover, adequate blood pressure reduction, cessation of cigarette smoking and the use of antithrombotic therapy in atrial fibrillation are the most effective modifiable risk factors in stroke prevention [29].

### Influence of age

Age has been identified as a marker of risk for stroke [30]. From the current trend it appears that both developed and developing countries are watching a rise in the population of old people, a number which is expected to increase over the coming decades. It is estimated that by 2050 there will be 56.9 million nonagenarians worldwide, an 800% increase compared with the situation prevalent today. It is within this group, that the prevalence and incidence of stroke is very high, greatly impacting morbidity and mortality [30]. In Saudi Arabia the frequency of stroke showed a steady increase with age until the 7th decade [28]. Recently, Al-Jadid reported that stroke occurred with a higher frequency in the 61-70 age group, and with a lower frequency in the 20-30 and 31-40 age groups [13]. Another study from Saudi Arabia also reported that stroke occurred most frequently in the 61-70 age group, while those least affected fell in the 30-40 age group [31].

### Gender differences

Epidemiological studies, mainly based on Western European surveys, have reported that compared with women, stroke strikes men more often, the incidence being about 30% higher [32]. For cerebral infarction the excess was 45%, with very little variance between the sexes, when considering intracerebral hemorrhage. For subarachnoidal hemorrhage, the association between the sexes was reversed, with a male deficit of about 50% [33]. In recent years, sex-specific data regarding stroke incidence, its prevalence, subtypes, severity and case-fatality from across the globe have also become readily available [32]. A few studies are available on the gender differences of stroke in Saudi Arabia, indicating that there were more men with strokes of all types than women [24]. Another study also reported that male patients formed the higher risk group compared with the female ones [13]. A study conducted on 500 Saudi patients with stroke indicated 68.4% were males and 31.6% were females [17].

### Neuropsychiatric manifestations

Depression, one of the most common neuropsychiatric manifestations in acute stroke, was revealed in 6-52% of stroke survivors [34, 35]. Associated with a delay in physical and mental recovery, depression has been found to lead to increase morbidity. In Saudi Arabia a distinct lack of data regarding depression following acute stroke has been observed. However, recently Hamad et al., (2011) reported that in a study conducted on 60 patients, the distribution of depression in 10 patients (17%) revealed a mild depression in 7 patients, moderate in 2, and severe in one patient [36]. They further reported that depression was observed only infrequently in the Saudi cohort following acute stroke, and was considerably related to the severity of disability, although not to the stroke type or site [36].

### Health-related quality of life

Health-Related Quality of Life (HRQOL) which covers mainly the physical, cognitive and social functions has been emphasized as an imperative index of the outcome after stroke; therefore, assessing the HRQOL assumes great importance for a stroke survivor [37, 38]. Numerous factors including age, gender and dependency for the Activities of Daily Living (ADL) / disability, and decreased social support have been connected with a low HRQOL value in a stroke survivor. Many studies have reported that the Quality of Life (QOL) in stroke patients is much lower than the QOL of the general population during the first few years after the stroke, particularly with respect to the physical factors [37, 38]. The QOL of Saudi stroke patients is generally low compared with those of some developed countries. A study done in Saudi Arabia reported that age and functional status strongly influenced the HRQOL [39]. Further, they reported that the Mini-Mental State Examination (MMSE) and Functional Independence Measure (FIM) scores were significantly correlated with the Stroke Impact Scale-16 (SIS-16) [39].

### Length of hospital stay

Studies reported that age, gender, race and medical complications of stroke can increase the Hospital Length of Stay (HLoS) of stroke patients. Furthermore, the presence of hypertension, diabetes or heart disease may all adversely affect the functional outcome and HLoS of stroke patients [40, 41]. However, some studies have reported conflicting statistics as the populations studied were very often different. Besides, stroke severity is a strong and reliable predictor of HLoS [40, 41]. A recent hospital-based study from Saudi Arabia reported that the mean HLoS on the stroke rehabilitation program was 45 days. Further reports indicated that the HLoS of stroke patients increases with age. The HLoS of the 20-30 age group was 36 days, whereas it was 53 days for the 71-80 age group. However, a slight decrease was observed in the 81-90 age (50 ± 2.4) as compared with the 71-80 age group [13]. Also, they reported that the HLoS of Saudi males was longer than that of the females for all age groups [13].

Research has revealed that medical complications such as the presence of hypertension or diabetes mellitus may adversely affect the functional outcome and HLoS of stroke patients [40]. Certain studies have reported conflicting statistics because of the variety in the populations studied. However, stroke severity and the nature of stroke are strong and reliable predictors of HLoS [40]. Recently in Saudi Arabia, Al-Eithan et al., reported that for patients with left hemiplegia / hemiparesis the HLoS was 43.5 days, whereas patients with right hemiplegia/hemiparesis showed a HLoS value of 47.3 days. Compared with only stroke, patients suffering with stroke combined with diabetes mellitus and hypertension showed a significantly higher HLoS in patients with right and left hemiplegia/hemiparesis [31].

### Medical care and rehabilitation

Neurorehabilitation targets to hasten or speed up the patient ‘s independence and QOL by maximizing the ability and participation. WHO defines neurorehabilitation as “an active process by which those disabled by injury or disease achieve a full recovery or, if a full recovery is not possible, realize their optimal physical, mental and social potential and are integrated into their most appropriate environment”. It is extremely essential to begin rehabilitation as soon as possible, post stroke onset [42]. Over the past two decades, the Ministry of Health (MOH), Saudi Arabia has established several rehabilitative services for persons with disabilities and other residents throughout the country. However, a majority of these programs offer only physical, occupational, speech and hearing therapy besides prosthetic and orthotic services within the existing modern and sophisticated health care service system and infrastructure [43, 44]. Rehabilitation programs and facilities, as an integral part of modern health care delivery services, have received due attention by government authorities, with high quality services being made available to all citizens and residents. In the beginning of the twenty-first century a few medical rehabilitation centers were introduced in the MOH hospitals. In addition, some private non-profit centers, such as Sultan Bin Abdulaziz Humanitarian City were started [43–45]. Presently, there are several rehabilitation hospitals / centers available in Saudi Arabia, mainly in the large cities, such as the Rehabilitation Unit of Prince Sultan Military Medical City of Riyadh, Rehabilitation Unit of King Abdulaziz Medical City, National Guard (Riyadh), Rehabilitation Hospital of King Fahad Medical City (Riyadh), King Saud Medical Complex, Rehabilitation Hospital of Al-Hada Military Hospital (Taif) and Riyadh Care Hospital [43–45]. However, stroke care in Saudi Arabia is yet to reach the levels of developed countries with only one active stroke center and seven centers providing thrombolysis, out of more than 350 hospitals nationally; only two hospitals have a stroke team with implemented triaging pathways and a beeper system. Establishing stroke units, increasing public awareness, training health care providers and collaboration are essential and urgent needs to be met as early as possible in Saudi Arabia [46]. Also, recent studies reported that there is a great demand for more rehabilitation centers, rehabilitation medicine physicians and a team of rehabilitation professionals in the Kingdom of Saudi Arabia [44, 47, 48].

## Conclusion

In Saudi Arabia, there is definite lack of published researches on stroke. However, such research is vitally essential to plan for appropriate management programs to be set up, effective implementation of primary prevention strategies and proper allocation of health resources in this area.
